# Editorial on Special Issue “Advances in Natural Products for Cutaneous Application”

**DOI:** 10.3390/pharmaceutics17050639

**Published:** 2025-05-12

**Authors:** Ana Žugić, Vanja Tadić, Ivana Nešić

**Affiliations:** 1 Department of Pharmaceutical Research and Development, Institute for Medicinal Plant Research “Dr. Josif Pancic”, 11000 Belgrade, Serbia; vtadic@mocbilja.rs; 2 Department of Pharmacy, Faculty of Medicine, University of Nis, 18000 Nis, Serbia; ivana.nesic@medfak.ni.ac.rs

## 1. Introduction

A growing trend of “returning to nature”, and a subsequent increase in the popularity of plant-based products, has arisen as a result of the activities of environmental movements around the world and emerging evidence about the side effects of synthetic substances. Research has revealed that a complex mixture of the active ingredients derived from crushed or powdered plant parts, such as resins, medicinal fats, essential oils, or plant extracts, may target several parts of the human body simultaneously, making these herbal preparations important for maintaining the health, prevention, and treatment of various conditions [1,2,3,4].

Topical use of plants for the care of normal or treatment of impaired skin stretches back millennia, in the form of ointments, decoctions, compresses, and poultices, mostly intended to treat wounds, hemorrhoids, boils, and eczema [5,6,7]. As anticipated, aside from the increased usage of plant-based products for cutaneous application, the stated trend of “returning to nature” has led to increased scientific interest in investigating natural bioactive compounds (NBCs) either as isolated compounds or as part of complex mixtures in plant isolates.

With this in mind, the Special Issue “Advances in Natural Products for Cutaneous Application” was intended to highlight the specificities of this type of natural product and to determine the latest trends related to this research area. Aside from the typical requirements for all cosmetic and medicinal products, such as safety demands, specific requirements such as satisfactory organoleptic and sensory properties of the formulation aimed at providing beneficial skin effects and/or adequate skin penetration of active substance are of particular importance for products intended for cutaneous application. Incorporating herbal extracts as active substances in dermal products imposes additional requirements, such as the identification and quantification of active or marker substances in these compounded mixtures of chemical entities. Furthermore, the stability of these complex products should be assessed in order to fulfill certain quality criteria.

## 2. Overview of the Published Articles

The 10 papers published in this Special Issue collectively highlight advances in cutaneous application of NBCs, either as isolated compounds or as part of plant extracts, encompassing recent research trends. The collected manuscripts address several topics: 1. encapsulation of NBCs into contemporary carriers, 2. healing potential of NBC-based formulations in the treatment of diabetic wounds, 3. usage of crop-derived NBCs/extracts in cosmetic and/or dermal products, 4. application of fermented or enzyme-assisted extracts in cutaneous products, and 5. novel herbal-based treatment options for atopic dermatitis.

Two papers in this Special Issue explore liposomes encapsulation of NBCs for potential cutaneous application. In one paper (contribution 7), ascorbyl palmitate, a derivative of L-ascorbic acid, was encapsulated into liposomes which were subsequently incorporated into cream and emulgel (2%). Encapsulation led to a significant total amount of penetrated ascorbyl palmitate in the *stratum corneum* (1.3-fold and 1.2-fold for the emulgel and cream, respectively). In the other paper, encapsulation of *Paeonia tenuifolia* L. (steppe peony) petal extract was achieved, followed by comprehensive physicochemical and biopharmaceutical characterization. Bearing in mind the high encapsulation efficacy of the extract’s NBCs into Phospholipon-based liposomes that enabled their slower release, as well as efficient inhibition of both bacterial and fungal growth and bacterial biofilm formation, the authors concluded that the developed liposomes may be used in creams and butters with potential beneficial skin-effects (contribution 2).

Three manuscripts in this Special Issue were devoted to the in vivo investigation of natural products (immortelle and Siberian pine essential oils, as well as pine tar) incorporated into simple vehicles for the potential treatment of chronic diabetic wounds. These reports offer an improved understanding of the wound-healing properties of natural products with a long history in traditional medicine, employing an excision wound model in diabetic *Wistar albino* rats. In addition, the results of the studies suggested these products’ potential as novel wound-healing agents. Considering the in vivo model used, the studies were also scientifically supported in their prospective application in chronic diabetic wound treatment, which represented a global health issue and social burden (contributions 1, 6 and 10).

Two papers within the Special Issue dealt with the pertinent topic of natural and sustainable approaches to skincare and healthcare using multipurpose crops, or their waste, as raw materials in products for cutaneous application. Thus, one investigation focused on the potential of maize, wheat, and sunflower waste ethanolic and lipid extracts as potential versatile raw materials for the development of topical products. The tested extracts had a satisfactory safety profile, contributed to the microbiological quality, and enhanced the emollient effect, leading to increased skin hydration, whilst simultaneously affecting sensory properties such as smell and color, the features that necessitated additional testing of the investigated formulations (contribution 8). In the other review article, the potential of hemp, a highly attractive environmentally friendly crop and an absolute champion of bioeconomy, was investigated to gain greater insight into its potential cutaneous application. However, even though hemp-based products were proven to be applicable for skin care and for the treatment of various dermatological conditions, regulatory considerations regarding hemp, which are often inconsistent globally and may be country-specific, were found to be of utmost importance when developing these products (contribution 5).

As herbal-based products are in high demand among consumers, who wish to use them for care and/or treatment of skin, the cosmetic and pharmaceutical industries are constantly searching for innovative approaches that can be used to achieve their active principles, e.g., either isolated NBCs or plant extracts. Regarding the latter, these include, among others, transformations of plant extracts by bacteria or fungi, by which the fermented plant extracts (FPEs) are obtained. In this vein, the topic of FPEs was described in one review article within this Special Issue, providing readers with detailed insights into their various biological activities, including their antimicrobial, antioxidant, anti-inflammatory, anti-melanogenic, and wound-healing properties. Further, the article described the use of FPE-based products for cutaneous application, such as anti-aging and anti-photoaging, anti-wrinkle, skin whitening, moisturizing products, hair growth products, products for androgenic or diffuse alopecia treatment, and wound-healing products. The manuscript highlighted the great potential of FPEs connected to their enhanced biological activity due to the significant increase in bioactive compounds under the action of microbial enzymes. However, it also emphasized the limitations associated with wider usage of FPEs related to their qualitative and quantitative chemical composition, including the possible generation of methanol, formaldehyde, biogenic amines, and nitrite during fermentation, as well as during storage stability (contribution 3). A similar approach that involved obtaining extract containing a higher amount of biologically active compounds using a commercially available blend of enzymes produced by microorganisms (which are not present in the blend, unlike in enzymatic transformations during the production of FPEs) was described in one paper in this Special Issue. The paper (contribution 9) dealt with enzymatically derived blackcurrant extract with high antioxidant activity, which was successfully incorporated into three vehicles (hydrogel, o/w cream gel, and o/w emulsion). Thus, hydrogel was shown to be a suitable carrier for the topical delivery of anthocyanins from blackcurrant, while their dermal and transdermal delivery was very limited, implying the potential applicability of the developed formulation for the targeted treatment of the skin surface (including, for instance, prebiotic or photoprotective effects).

Lastly, one paper (contribution 4) was dedicated to the ever-relevant topic of atopic dermatitis. This manuscript investigated the influence of the herbal mixture LK5, composed of *Scutellaria baicalensis*, *Liriope platyphylla*, *Sophora flavescens*, *Dictammus dasycarpus*, and *Phellodendron schneid,* on the activation of the JAK-STAT pathway responsible for the increase in the expression of inflammatory cytokines such as IL-4 and IL-13, thus deteriorating atopic dermatitis. The results of this promising study suggest that LK5 might modulate the immune response and alleviate AD symptoms by inhibiting STAT pathways.

## 3. Conclusions/Future Directions

Overall, as evidenced by the published studies, advances in natural products for cutaneous application have informed contemporary strategies for enhancing the biological activities of herbal-based products for skin application, including encapsulation and usage of plant extracts obtained via novel extraction approaches. Also, the application of medicinal plants for the treatment of wounds from traditional medicine has been justified by several papers, while new herbal-based options for the treatment of atopic dermatitis, as one of the most common skin impairments of the present, have been proposed. Lastly, the studies included in this Special Issue have opened up the topic of using industrial crops, or the associated waste products, as raw materials for the cosmetic/pharmaceutical industry. Future research on this topic should include additional investigations that would translate the findings of the aforementioned studies into clinical practice and/or everyday use.

## Data Availability

The authors declare no conflicts of interest.
